# Hypoglycemia following intravenous insulin plus glucose for hyperkalemia in patients with impaired renal function

**DOI:** 10.1371/journal.pone.0172961

**Published:** 2017-02-28

**Authors:** Armando Coca, Ana Lucia Valencia, Jesus Bustamante, Alicia Mendiluce, Jürgen Floege

**Affiliations:** 1 Department of Nephrology, Hospital Clínico Universitario, Valladolid, Spain; 2 Department of Medicine, Dermatology and Toxicology, School of Medicine, University of Valladolid, Valladolid, Spain; 3 Department of Nephrology, RWTH University of Aachen, Aachen, Germany; University of Sao Paulo Medical School, BRAZIL

## Abstract

**Background:**

Hypoglycemia is a serious complication following the administration of insulin for hyperkalemia. We determined the incidence of hypoglycemia and severe hypoglycemia (blood glucose <70 or ≤40 mg/dl, respectively) in a cohort of AKI and non-dialysis dependent CKD patients who received an intravenous infusion of insulin plus glucose to treat hyperkalemia.

**Methods:**

We retrospectively reviewed charts of all AKI and non-dialysis dependent CKD patients who received 10 U of insulin plus 50 g glucose to treat hyperkalemia from December 1, 2013 to May 31, 2015 at our Department.

**Results:**

One hundred sixty four episodes of hyperkalemia were treated with insulin plus glucose and were eligible for analysis. Serum potassium levels dropped by 1.18 ± 1.01 mmol/l. Eleven treatments (6.1%) resulted in hypoglycemia and two (1.2%) in severe hypoglycemia. A lower pretreatment blood glucose tended to associate with a higher subsequent risk of hypoglycemia. Age, sex, renal function, an established diagnosis of diabetes or previous treatment were not associated with the development of this complication. We did not register any significant adverse events.

**Conclusion:**

Our intravenous regimen combining an infusion of insulin plus glucose effectively reduced serum potassium levels compared to previous studies and associated a low risk of symptomatic hypoglycemia and other complications.

## Introduction

Hyperkalemia is a common electrolyte imbalance usually associated with reduced renal excretion, altered cation exchange at the cellular level and/or excessive intake. It has potential life-threatening consequences [1, 2] and increases all-cause and in-hospital mortality [3–5].

The primary goal of the management of hyperkalemia is the removal of excess K^+^. In end-stage renal disease (ESRD) emergency hemodialysis is the therapy of choice, while in patients with acute kidney injury (AKI), non-dialysis dependent chronic kidney disease (CKD) or normal renal function treatment should be directed at increasing K^+^ excretion via diuretics such as furosemide or ion-exchange resins such as calcium polystyrene sulfonate [6]. Because the initiation of the treatment of choice often involves a considerable delay, other more immediate measures must also be considered, such as promoting the shift of K^+^ from the extracellular to the intracellular compartment through the use of intravenous insulin plus glucose [7].

The binding of insulin to its receptor on the cell membrane of skeletal muscle stimulates the activity of Na^+^-H^+^ antiporter and increases both the cell membrane density and activity of Na^+^-K^+^ adenosine triphosphatase (ATPase) [6, 8, 9]. The activity of the Na^+^-H^+^ antiporter causes a flux of Na to the intracellular space, leading to the activation of the Na^+^-K^+^ ATPase and an influx of K^+^ from the extracellular compartment [10].

Hypoglycemia is a common complication following the use of glucose and insulin for hyperkalemia [7, 10, 11]. Its incidence in this setting ranges from 8.7% to 75% [12–15]. AKI, CKD and ESRD patients are particularly at risk of hypoglycemia [16–17]. Hypoglycemia mainly originates from an inadequate supply of glucose to compensate the reduction of blood glucose induced by the exogenous insulin. Renal injury may additionally aggravate hypoglycemia through decreased insulin degradation and gluconeogenesis [18, 19].

There is no consensus on what is the best and safest way to administer insulin in the treatment of hyperkalemia. The American Heart Association (AHA) guidelines [20] and the European Resuscitation Council Guidelines [21] recommend 10 U regular insulin and 25 g glucose intravenous over 15 to 30 minutes, but in studies insulin dosage ranged from 5 to 10 U and glucose from 25 to 60 g [22]. Other protocols include a second dose of 25 g glucose intravenously 60 minutes after the administration of the AHA guideline regimen to prevent hypoglycemia [13], the use of insulin aspart instead of insulin regular with a variable dose of glucose [14], the administration of a bolus of 5 U of insulin to be repeated every 15 minutes plus a 50 g/h glucose infusion [23], the use of a higher amount of insulin and glucose over a longer period of time [24] or even an insulin-free regimen with only a glucose bolus as treatment [25].

A standardized protocol has been used at our Department for hyperkalemia for over 10 years. The regimen includes a continuous intravenous infusion of 10 U of short-acting insulin (Insulin Actrapid, Novo Nordisk, Bagsværd, Denmark) and 50 g glucose (500 ml of 10% dextrose) over 240 minutes plus 40 mg furosemide. It was administered to all AKI and non-dialysis dependent CKD patients with hyperkalemia without contraindications for it (e.g. allergy to components). The protocol included quantification of blood glucose and K^+^ four hours after the end of the infusion. Additional blood glucose including point-of-care and K^+^ values measured at the discretion of clinicians during the first eight hours after the infusion were also included. We conducted a retrospective study to assess the incidence of hypoglycemia in the treatment of hyperkalemia in patients with AKI, defined by an increase in absolute serum creatinine of at least 0.3 mg/dl or by a percentage increase in serum creatinine ≥ 50% (1.5 times baseline value) [26] and non-dialysis dependent CKD. Emergent dialysis was considered the treatment of choice in dialysis dependent patients. Loop diuretics such as furosemide would be ineffective in most patients in this setting, the rate of fluid administration would be limited by the degree of fluid overload of each subject, and the intravenous infusion would be stopped as soon as dialysis was initiated, thus hindering the administration of our regimen in a homogenous and controlled manner. Therefore, dialysis dependent were not considered for this analysis.

## Materials and methods

### Study design

We performed a retrospective, single-center review of all adult subjects with AKI or non-dialysis dependent CKD and a serum K^+^ of ≥ 6 mmol/L who received our standardized intravenous regimen to treat hyperkalemia from December 1, 2013 to May 31, 2015. We included all patients aged 18 or older who had a documented order for the regimen, a serum K^+^ concentration ≥ 6 mmol/l and a registered blood glucose value within 6 hours prior to the administration of the infusion. Patients without a documented blood glucose value and serum K^+^ concentration 4 hours after the end of the infusion were excluded. All patients received the standardized intravenous regimen.

We collected information from the hospital’s electronic database, including sex, age, race, diagnosis of diabetes, previous treatment and analytical parameters such as kidney function, blood glucose and serum K^+^. Kidney function was assessed at two different time points: preadmission and during the episode. Glomerular filtration rate (eGFR) was estimated using the Modification of Diet in Renal Disease (MDRD) formula [27]. The study was approved by the institutional review board and ethics committee of our hospital (Comité Ético de Investigación Clínica del Área de Salud de Valladolid Este, Hospital Clínico Universitario de Valladolid) and was performed in accordance with the ethical principles of the Declaration of Helsinki. The ethics committee ruled that informed consent did not apply due to the observational and retrospective nature of the study.

### Study end points

The primary endpoint was the development of hypoglycemia, defined as a blood glucose < 70 mg/dl in the study population. All blood glucose values were collected within 8 hours of the end of the intravenous infusion. The lowest blood glucose within this time period was included for each patient in the analyses. Secondary endpoints included the incidence of severe hypoglycemia, defined as a blood glucose ≤ 40 mg/dl and percentage change of serum K^+^ within the time period.

### Statistics

Descriptive statistics were used to determine incidence rates and percentages at the patient level. We used mean and standard error for continuous variables or frequency counts or percentages for categorical variables. We performed a *χ*^2^ test or Fisher’s exact test to assess differences for categorical variables between study groups. Student’s t-test or Wilcoxon rank-sum test were used for continuous variables. Bivariate logistic regression was used to determine associations between variables. The statistical analysis was performed using IBM SPSS Statistics Version 22 (Chicago, IL, USA).

## Results

### Epidemiology and timing of hypoglycemia

**Fig 1:**
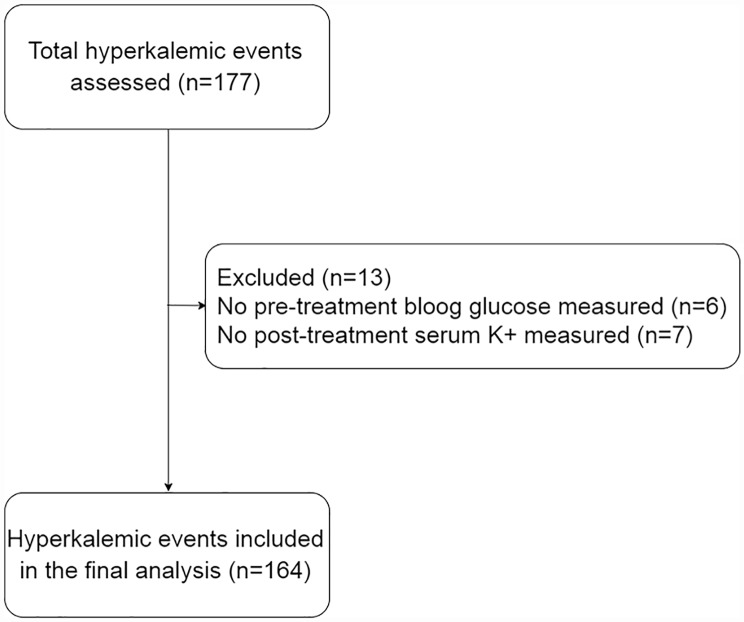
Flow diagram.

**Fig 2:**
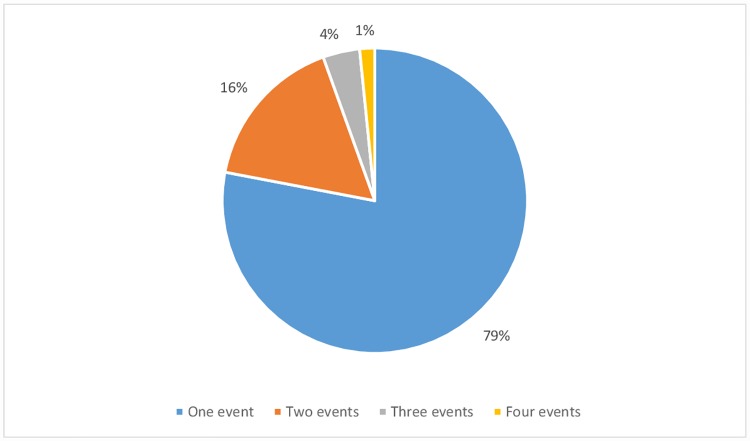
Hyperkalemic events per patient.

We identified 177 episodes where an intravenous infusion of insulin and glucose was administered to treat hyperkalemia. One hundred sixty four episodes of hyperkalemia complied with the inclusion and exclusion criteria and were included in the final analysis (Fig 1). These episodes occurred in a total of 127 patients. All patients were Caucasians. Fifty-three (41.7%) of the 127 patients had diabetes. One hundred twenty-nine episodes occurred in patients with AKI (including acute exacerbations of CKD) while 35 episodes occurred in CKD patients. Twenty-three episodes occurred in patients with a preadmission eGFR ≥ 60 ml/min while 141 episodes occurred in patients with a preadmission eGFR < 60 ml/min. A single laboratory measurement of blood glucose and serum K^+^ was performed in 145 episodes (88.4%), two measurements in 16 episodes (9.8%) and three measurements in 3 episodes (1.8%). Follow-up of associated pathologies such as anemia or metabolic acidosis was the underlying cause of additional laboratory measurements. There were no significant differences in the number of laboratory measurements between patients with a blood glucose pretreatment level ≥ 120 mg/dl or lower than that value [N° measurements (pretreatment blood glucose < 120 mg/dl): 1.1 ± 0.3; N° measurements (pretreatment blood glucose ≥ 120 mg/dl): 1.17 ± 0.46; P = 0.25]. The majority of patients (78%) experienced only a single episode of hyperkalemia (Fig 2). No patient who experienced more than one episode of hyperkalemia suffered multiple episodes of hypoglycemia.

An average decline of 1.18 ± 1.01 mmol/l of serum K^+^ concentration was attained after treatment. The efficacy of the regimen in reducing serum K^+^ levels did not differ between hypoglycemic or non-hypoglycemic patients, nor between patients with a preadmission eGFR higher, equal or lower than 30 ml/min.

Ten (6.1%) of the 164 hyperkalemia episodes resulted in hypoglycemia. Of these patients, four (2.4%) presented symptomatic hypoglycemia with point-of-care glucose <70 mg/dl. Eight patients (4.9%) developed symptoms suggestive of hypoglycemia but presented a point-of-care glucose value >70 mg/dl. Blood glucose was between 41–70 mg/dl in eight episodes (4.9%) and equal or lower than 40 mg/dl in two episodes (1.2%). Hypoglycemic events occurred at an average time of 3.5 hours after the end of the intravenous infusion. Both episodes of severe hypoglycemia occurred in the first 3 hours after the intravenous infusion. Only one episode of hypoglycemia happened after the sixth hour after the end of the intravenous infusion.

### Risk factors

**Fig 3:**
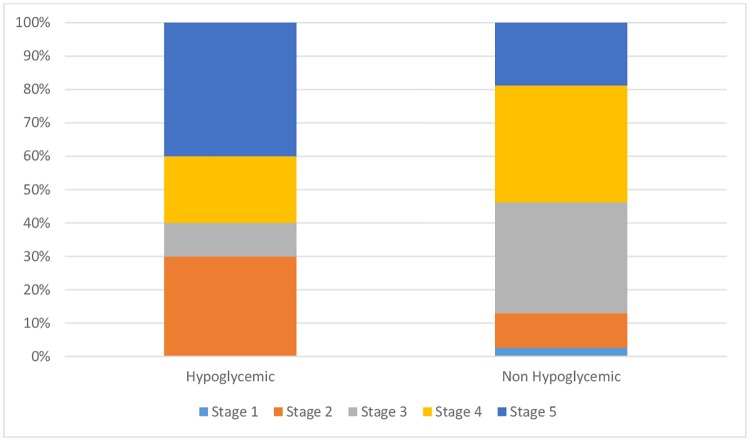
Preadmission CKD stage distribution comparison between groups.

There were no significant differences in age, sex, preadmission renal function, current renal function (Fig 3) or established diagnosis of diabetes between the groups with or without hypoglycemia (Table 1). Patients with a pretreatment blood glucose level lower than 120 mg/dl were more likely to develop hypoglycemia, although this association was not significant in our sample (Table 2). Hypoglycemic patients also exhibited a significantly higher mean decrease of blood glucose levels than non-hypoglycemic patients (Table 1). There were no significant differences in pretreatment K^+^, mean decrease of serum K^+^ or nadir K^+^ levels between patients with hypoglycemia compared to those without hypoglycemia (Table 1). Episodes associated with CKD or AKI presented no differences regarding mean K+ decrease (CKD: 1.1 ± 0.97 mmol/l, AKI: 1.21 ± 1.02 mmol/l; P = 0.58) or mean blood glucose decrease (CKD: 14.7 ± 100.5 mg/dl, AKI: -1.4 ± 72.8 mg/dl; P = 0.28). We also found no difference between episodes with preadmission eGFR < 60 ml/min or ≥ 60 ml/min regarding mean K^+^ decrease (eGFR <60 ml/min: 1.16 ± 0.99 mmol/l, eGFR ≥60 ml/min: 1.33 ± 1.05 mmol/l; P = 0.43) or mean blood glucose decrease (eGFR <60 ml/min: 1.8 ± 82.4 mg/dl, eGFR ≥60 ml/min: 3.3 ± 60.2 mg/dl; P = 0.94).

**Table 1:** Comparison of patient characteristics between those with or without hypoglycemia following the administration of the standardized protocol.

	Blood glucose < 70 mg /dl (n = 10)	Blood glucose ≥ 70 mg/dl (n = 154)	P Value
**Age, years [mean ± SD]**	72 ± 12	75 ± 11	0.41
**Males, n (%)**	6 (60)	89 (57.8)	0.89
**eGFR (preadmission), ml/min/1.73m**^2^ **[mean ± SD]**	34.3 ± 32.4	33.7 ± 24.2	0.94
**eGFR (current), ml/min/1.73m**^2^ **[mean ± SD]**	17.4 ± 17.2	15.3 ± 17.2	0.7
**Diabetes mellitus, n (%)**	5 (50)	66 (42.9)	0.65
**Blood glucose (pretreatment), mg/dl [mean ± SD]**	111.2 ± 44.8	152.3 ± 80	0.11
**Blood glucose (nadir), mg/dl [mean ± SD]**	53 ± 14.4	153.9 ± 81.2	**<0.001**
**Blood glucose (mean decrease), mg/dl [mean ± SD]**	58.2 ± 45.2	-1.6 ± 79.9	**0.021**
**Serum K**^+^ **level (pretreatment), mmol/l [mean ± SD]**	6.79 ± 0.9	6.85 ± 0.8	0.78
**Serum K**^+^ **level (nadir), mmol/l [mean ± SD]**	5.42 ± 0.7	5.68 ± 0.8	0.32
**Serum K**^+^ **(mean decrease), mmol/l [mean ± SD]**	1.37 ± 0.88	1.17 ± 1.01	0.54
**Receiving insulin, n (%)**	3 (30)	34 (22.1)	0.68
**Receiving oral ADAs, n (%)**	3 (27.3)	35 (21)	0.56
**Receiving ACEI or ARB, n (%)**	5 (50)	89 (57.8)	0.63
**Receiving β-blocker, n (%)**	3 (30)	43 (27.9)	0.88
**Receiving loop diuretic, n (%)**	3 (30)	84 (54.5)	0.13
**Receiving K**^+^ **sparing diuretic, n (%)**	1 (10)	29 (18.8)	0.48
**Receiving NSAID, n (%)**	1 (10)	46 (29.9)	0.18

**Table 2:** Bivariate analysis of the association between hypoglycemia and different patient characteristics.

	OR (95% CI)	P Value
**Female compared to male**	0.91 (0.24–3.36)	0.8
**Age ≥ 75y compared to < 75y**	0.9 (0.25–3.24)	0.9
**Diagnosis of diabetes compared with no diabetes**	1.33 (0.37–4.79)	0.7
**Pretreatment glucose level < 120 mg/dl to ≥ 120 mg/dl**	4.44 (0.91–21.57)	0.055
**Preadmission GFR < 30 compared to ≥ 30**	1.28 (0.34–4.72)	0.75
**Current GFR < 30 compared to ≥ 30**	1.28 (0.35–4.73)	0.75
**Insulin users compared to non-users**	0.71 (0.15–3.28)	0.9
**Oral antidiabetic drug users compared to non-users**	1.51 (0.37–6.12)	0.69
**ACEI or ARB users compared to non-users**	0.73 (0.2–2.63)	0.74
**β-blocker users compared to non-users**	1.11 (0.27–4.48)	0.9
**Loop diuretic users compared to non-users**	0.36 (0.09–1.43)	0.19
**K**^+^ **sparing diuretic users compared to non-users**	0.47 (0.06–3.93)	0.69
**NSAID users compared to non-users**	0.26 (0.03–2.11)	0.43

**GFR**: Glomerular filtration rate, ml/min/1.73m2. **ACEI**: Angiotensin-converting-enzyme inhibitor. **ARB**: Angiotensin II receptor blocker. **NSAID**: Nonsteroidal anti-inflammatory drug.

### Role of diabetes

The mean pretreatment and nadir blood glucose levels were significantly higher in patients with a prior diagnosis of diabetes as compared to non-diabetic patients. There were no significant differences in pretreatment or nadir K^+^ levels, preadmission or current eGFR between diabetic and non-diabetic patients (Table 3). No significant difference was found in mean K^+^ decrease between episodes with pretreatment blood glucose ≥200 mg/dl (1.42 ± 1.17 mmol/l, N = 34) compared to episodes with pretreatment blood glucose <200 mg/dl (1.12 ± 0.95 mmol/l, N = 130; P = 0.16).

**Table 3:** Comparison of analytical parameters between diabetics and non-diabetics.

	Non diabetics (n = 93)	Diabetics (n = 71)	P Value
**Blood glucose (pretreatment), mg/dl**	122.1 ± 41.6	186.2 ± 99.2	**<0.001**
**Blood glucose (nadir), mg/dl**	127.4 ± 57.7	174.5 ± 100.7	**<0.001**
**Serum K**^+^ **(pretreatment), mmol/l**	6.86 ± 0.8	6.84 ± 0.7	0.83
**Serum K**^+^ **(nadir), mmol/l**	5.66 ± 0.8	5.68 ± 0.9	0.88
**eGFR (preadmission), ml/min/1.73m**^2^	34.9 ± 26.9	32.2 ± 21.2	0.49
**eGFR (current), ml/min/1.73m**^2^	14 ± 19.9	17.2 ± 12.5	0.23
**Blood glucose (mean decrease), mg/dl**	-5.3 ± 58.4	11.7 ± 100.3	0.21
**Serum K**^+^ **(mean decrease), mmol/l**	1.2 ± 1.01	1.15 ± 0.99	0.77

### Associated adverse events

We studied the development of adverse events during the intravenous infusion administration and the 8-hour follow up period. Besides symptomatic hypoglycemia we did not observe other adverse clinical events such as dysrhythmias, cardiovascular events or pulmonary edema.

## Discussion

Hypoglycemia is an often under-appreciated problem and a common inpatient complication. Diabetic patients who develop hypoglycemia are at a six-fold increased risk of death compared to diabetics who do not experience this complication [28]. Here we have retrospectively analyzed a large group of patients with acute or chronic kidney disease (Table 4) to assess the development of hypoglycemia following an intravenous infusion of insulin plus glucose to treat an episode of hyperkalemia. Our protocol includes the administration of insulin plus glucose by continuous intravenous infusion and thus differs from most published studies which usually opt for bolus administration (Table 4). Our protocol was specifically designed to avoid sudden changes in blood glucose levels while retaining the ability to reduce the level of serum K^+^. A second particular feature of our protocol is the use of rapid-acting insulin instead of regular insulin to prevent the longer lasting hypoglycemic effect of regular insulin.

**Table 4:** Comparison of clinical studies of insulin plus glucose for the treatment of hyperkalemia.

Reference	Present study	[28]	[28]	[25]	[25]	[13]	[12]	[14]	[15]
**Sample size**	**164**	71	78	10	10	219	221	86	12
**Kidney Function**	**AKI/CKD**	CKD/ESRD	CKD/ESRD	ESRD	ESRD	All	ESRD	All	ESRD
**Glucose dose (g)**	**50**	25	25	50	50	0–50	Variable	0–25	25
**Insulin dose (U)**	**10**	5	10	10	0	5–10	4–10	Variable	10
**Route of administration**	**Infusion**	Bolus	Bolus	Bolus	Bolus	Bolus	Bolus	Bolus	Bolus
Mean initial glucose*	**149**	-	-	100	91	149	154	150	88.2
Peak change in glucose*	**-2**	-	-	-5	47	-	-9	-	-37.8
**Mean initial K**^**+**^ **	**6.85**	6.3	6.3	6	6.2	-	-	-	5.5
**Peak reduction in K**^**+**^ **	**1.18**	1.08	1.10	0.83	0.50	-	-	-	0.65
**Follow-up time (hrs)**	**8**	8	8	1	1	>3	>3	-	1
Hypoglycemia limit*	**<70**	≤70	≤70	≤50	≤50	≤70	<60	<70	Undefined
**Hypoglycemia (%)**	**6.1**	19.7	16.7	20	0	8.7	13	17.4	75
Severe hypoglycemia limit*	**≤40**	<50	<50	-	-	<40	-	<40	-
**Severe hypoglycemia (%)**	**1.2**	7	8.9	-	-	2.3	-	3.5	-

* mg/dl;

** mmol/l

We observed an average decrease of serum K^+^ concentration of 1.18 mmol/l, which is of a very similar magnitude as that observed by others [29] in hyperkalemic patients with a low eGFR following the administration of a bolus of 5 or 10 U of regular insulin plus 25 g glucose (Table 4). In contrast, a bolus regimen of 50 g glucose plus 0 or 10 U regular insulin led to a much lesser reduction of serum K^+^ concentration (-0.5 and -0.83 mmol/l, respectively) in hyperkalemic patients on dialysis [25]. Thus, our regimen compares favorably in terms of effective lowering of serum K^+^ concentration with data in the literature (Table 4). Only a single small study in 22 non-CKD patients with hyperkalemia after the administration of a high-concentration K^+^ solution to perform continuous warm-blood cardioplegia reported a higher efficacy (-1.78 mmol/l K^+^) using a very high amount of glucose (100 g) and an even higher insulin bolus (50 U) [30]. This study was not included in Table 4 due to its different clinical setting.

The most extensively used regimen is that of the AHA, consisting of a bolus intravenous injection of 10 U of regular insulin plus 25 g of glucose [20]. It is known that a 500 μU/ml plasma insulin concentration is needed to achieve maximal insulin-mediated K^+^ uptake, while a much lower insulin concentration (100 μU/ml) is needed to attain maximal glucose uptake [6]. Based on this, one can estimate that the AHA regimen only reaches a high enough insulin concentration to effect maximal K^+^ uptake for 20 minutes while maintaining maximal glucose uptake for an additional 40 minutes, thus resulting in excess hypoglycemic capacity. It has been shown that it would be necessary to infuse 40 g of glucose per hour to maintain serum glucose levels within the normal range at a maximal K^+^ uptake insulin concentration [31, 32]. While our treatment protocol may be able to lower serum K^+^ levels through an extended period of time, it is quite possible that a 4-hour infusion of 10 U insulin may not be able to reach a high enough plasma concentration to attain the maximal K^+^ uptake effect of the hormone. The use of concomitant medication to lower serum K^+^ levels, such as loop diuretics in patients with conserved urine output is important to ensure the decline of the serum concentration of K^+^, especially taking into account the lower K^+^ reuptake ability of muscle cells in the presence of elevated serum concentrations of this electrolyte.

The incidences of hypoglycemia and severe hypoglycemia in our study were 6.1% and 1.2%, respectively. While we used a lower serum glucose value to define severe hypoglycemia (≤40 mg/dl) than other studies (Table 4), its incidence rate did not change significantly when we raised the threshold value to <50 mg/dl. We monitored serum glucose levels for up to 8 hours. It has been described that hypoglycemic events can occur as long as 7.5 hours after insulin administration [29]. Monitoring hypoglycemic events for a shorter period of time may underestimate the incidence of this complication. Of note, despite our long period of observation, the incidence of hypoglycemia events in our study was lower than that reported by almost all other previous studies [12–15, 29] (Table 4). This may relate to our treatment regimen, which included 50 g of glucose, i.e. a higher dose than that given in most previous studies, and a 10 U dose of short-acting insulin infused over a 4-hour period. Both of this, i.e. high glucose and insulin infusion rather than bolus, will result in a high glucose/insulin ratio at any given time, which may protect from hypoglycemia. Indeed, a continuous intravenous insulin infusion has been linked to a decreased incidence of hypoglycemia when compared to a bolus injection of insulin [33]. Timing may be another critical issue, since a smaller dose of glucose or the administration of the insulin bolus before the infusion of glucose have been related to hypoglycemia events [25]. The use of short-acting insulin may be a third factor involved in the development of a low incidence of hypoglycemia in our study. A short-acting insulin (insulin aspart) has been associated to a shorter duration of action and a reduced effect of a decreased eGFR on the pharmacokinetics of the hormone [34]. A continued supply of glucose to the patient during the duration of action of the administered insulin might contribute to reduce the incidence of hypoglycemia even further.

In agreement with a previous study [13], patients who had a lower pretreatment blood glucose tended to have a higher risk of developing hypoglycemia, although this association did not reach statistical significance in our study. We found no association between a prior diagnosis of diabetes or the prior use of insulin or oral antidiabetic agents and the risk of hypoglycemia during the hyperkalemia treatment. Apel *et al* [13] described a borderline significantly higher risk for hypoglycemia in patients without a prior diagnosis of diabetes.

Age, sex or the use of different drugs, including ACEI/ARB, β-blockers, loop diuretics, K^+^-sparing diuretics and NSAID, were not significantly different between our patients with or without hypoglycemia. Although it has been described that patients with low eGFR are at an increased risk of developing hypoglycemia [13, 29], we did not observe significant differences between patients with an eGFR lower than 30 ml/min and the rest of the population.

Besides hypoglycemia we did not observe any adverse events in the patients enrolled in the study. Others described that adverse events, such as pulmonary edema, were associated with a glucose-only regimen [25].

Limitations of our study include its retrospective nature, which renders it difficult to control for all potentially confounding factors that may have influenced the results, such as urine output or time since last pre-admission administration of antidiabetic medication. Dialysis dependent patients were not considered for this study due to dialysis being the treatment of choice in those cases, hence our results should not be applied in that setting. We did not take into account potential additional doses of insulin, administered after the infusion period, although this possibility seems very unlikely in view of common treatment patterns at our hospital. Our protocol included quantification of blood glucose and K^+^ four hours after the end of the infusion plus additional point-of-care and laboratory measurements at the discretion of the clinician, but asymptomatic hypoglycemic events may have gone undetected, so the actual incidence of hypoglycemia might have been underestimated. We did not measure urinary potassium excretion or urine output during follow-up, which makes it difficult to differentiate the percentage of serum K^+^ change associated to urinary elimination. All patients included in the study were Caucasian, thus limiting ethnic generalizability of results.

## Conclusion

In summary, the treatment regimen proposed in this study carries a low risk of symptomatic hypoglycemia and other complications in patients with reduced eGFR, despite good efficacy to lower serum K^+^ levels as compared to other studies.
